# Small protein, big effects: ENOD93 alters mitochondrial ATP production to favor nitrogen assimilation in plants

**DOI:** 10.1093/plcell/koae247

**Published:** 2024-09-05

**Authors:** Renuka Kolli

**Affiliations:** Assistant Features Editor, The Plant Cell, American Society of Plant Biologists; Sainsbury Laboratory, University of Cambridge, Cambridge, UK

Mitochondria are referred to as the powerhouse of the cell, and in plants, they generate the majority of ATP needed to energize various cytosolic processes. Oxidative phosphorylation of ADP to ATP occurs when a proton motive force generated across the mitochondrial inner membrane by electron transfer through a series of respiratory chain complexes fuels the ATP synthase complex. Respiratory complex IV catalyzes the terminal electron transfer from cytochrome *c* to molecular oxygen, reducing it to water, and contributes to the proton gradient by pumping protons across the inner membrane. In yeast, a protein called Rcf2 supports the proton motive force generation by complex IV (Strogolova et al. 2019). However, its counterpart in plants was so far unknown.

Using hidden Markov models, **Chun Pong Lee and coauthors (Lee et al. 2024)** identified early nodulin 93 (ENOD93) as a homolog of the yeast Rcf2 N-terminal domain (Fig. 1). On the other hand, another plant protein, hypoxia-induced gene domain 2 (HIGD2), was found to be similar to the Rcf2 C-terminal domain (Fig. 1). Interestingly, Rcf2 is proteolytically processed into N- and C-terminal peptides in yeast (Fig. 1; Rompler et al. 2016). Therefore, the authors consider ENOD93 and HIGD2 as the N- and C-terminal domain equivalents of Rcf2, respectively. ENOD93, a small protein of about 12 kDa, is conserved in plants and many other eukaryotes. It is important for biological nitrogen fixation in legumes and nitrogen use efficiency in cereals (Bi et al. 2009; Yan et al. 2015). ENOD93 was predicted to be mitochondrial membrane-localized in those early studies, but its function remained unknown.

**Figure 1.**
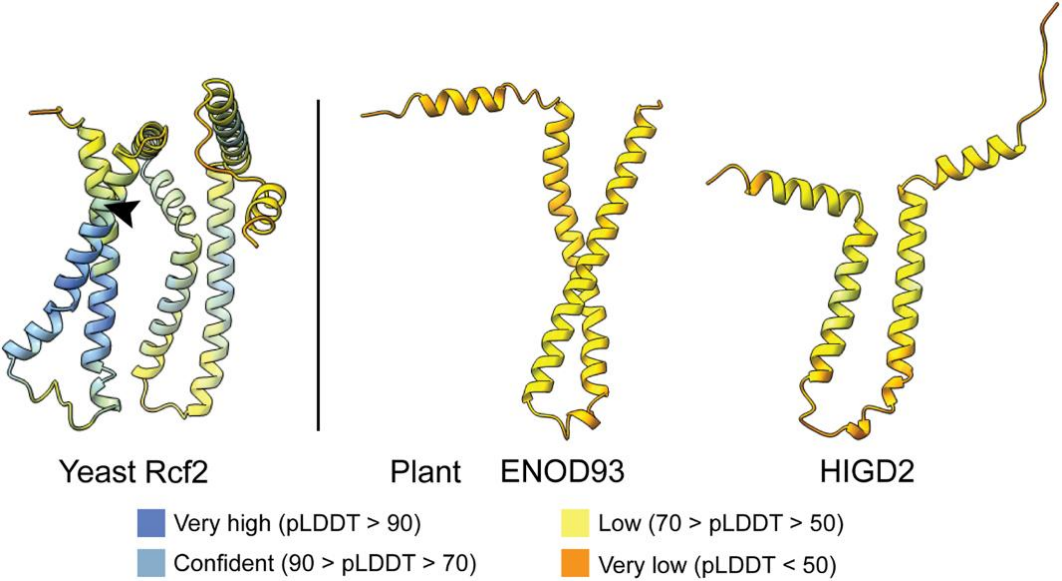
AlphaFold-predicted structures of Rcf2, ENOD93, and HIGD2 showing similarity between the yeast and plant homologs. The transmembrane regions are shown as vertical alpha helices. The putative cleavage site of Rcf2 is indicated with an arrowhead. The color code represents per-residue confidence score (pLDDT) values for the models. Figure credit: R. Kolli. The structures were obtained from the Uniprot database.

To investigate ENOD93 function, the authors performed physiological and biochemical characterization of an Arabidopsis (*A. thaliana*) *enod93* knockout mutant and its complemented lines expressing varying levels of ENOD93. The mutant displayed short roots and flowered early, while the complemented lines were similar to the wild type. Native complexome studies and 2-dimensional gel electrophoresis followed by peptide mass spectrometry indicated that ENOD93, like Rcf2, associates with complex IV. Complex IV abundance and native in-gel activity were unaffected in broken *enod93* mitochondria, but complex IV activity decreased in intact *enod93* mitochondria when electrons were directly passed to cytochrome *c* by providing a combination of N, N, N', N'-tetramethyl-p-phenylenediamine dihydrochloride and ascorbate. Hence, ENOD93 appears to impact complex IV activity in organello.

The authors found a more obvious decline in ADP-activated mitochondrial oxygen consumption rate of *enod93* when the membrane potential increased to a certain level. To test whether loss of membrane potential would recover the respiratory rate, the authors used FCCP, an ionophore that transports protons across the mitochondrial inner membrane, thus disrupting the proton gradient. Adding FCCP in oxygen-electrode assays recovered the declined oxygen consumption rates of *enod93* mitochondria to the levels observed in the wild type and an ENOD93 complemented line. Furthermore, the higher membrane potential maintained in *enod93* mitochondria, as measured by safranin fluorescence, reverted to a level similar to the wild-type and the complemented line when ferricyanide was used as an artificial electron acceptor to bypass complex IV activity. Despite the higher membrane potential, a major decline in ATP synthesis rate was observed in *enod93* mitochondria. Accordingly, the roots of *enod93* seedlings were found to have a lower ATP:ADP ratio than that in the wild type and the complemented lines. As the ATP:ADP ratio is an indicator of cellular energy status and regulates various metabolic activities, the lower ratio in *enod93* probably resulted in the observed short root phenotype.

Lee et al. have now uncovered the function of the enigmatic ENOD93, discovered over 3 decades ago, that it boosts mitochondrial ATP production by regulating complex IV activity. Supercomplex associations, kinetic coupling, or both between complex IV and ATP synthase are likely involved. The authors hypothesize that ENOD93 expression allows cells to meet the high ATP demand of nitrogen-fixing root nodules in legumes and of nitrogen uptake and nitrate reduction in cereals. Their findings serve as a baseline for future research on the regulation of plant mitochondrial oxidative phosphorylation. Studying the link between ENOD93 and HIGD2 is another interesting line of future research.

## Dive Curated Terms

The following phenotypic, genotypic, and functional terms are of significance to the work described in this paper:

FCCP CHEBI: CHEBI:75458

ADP CHEBI: CHEBI:16761

ATP CHEBI: CHEBI:15422
